# An unusual case of unilateral vascular hypoplasia in an adult patient – late diagnosis of PHACE syndrome

**DOI:** 10.1016/j.ijcchd.2023.100465

**Published:** 2023-06-30

**Authors:** Madelien V. Regeer, J. Lauran Stöger, Regina Bökenkamp, Inge M.M. Lakeman, Mark G. Hazekamp, Philippine Kiѐs, Anastasia D. Egorova, Monique R.M. Jongbloed

**Affiliations:** a CAHAL, Center for Congenital Heart Disease Amsterdam Leiden, Leiden University Medical Center, Leiden, the Netherlands; b Department of Cardiology, Leiden University Medical Center, Leiden, the Netherlands; c Department of Radiology, Leiden University Medical Center, Leiden, the Netherlands; d Department of Paediatric Cardiology, Leiden University Medical Center, Leiden, the Netherlands; e Department of Clinical Genetics, Leiden University Medical Center, Leiden, the Netherlands; f Department of Cardiothoracic Surgery, Leiden University Medical Center, Leiden, the Netherlands; g Department of Anatomy and Embryology, Leiden University Medical Center, Leiden, the Netherlands

**Keywords:** Embryology, (Double) aortic arch, Aberrant right subclavian artery, Vascular hypoplasia, Adult congenital heart disease, PHACE syndrome, Clinical genetics

## Abstract

A case of unilateral vascular hypoplasia is presented. A female patient was born with a complex aortic arch anatomy - a double aortic arch with an interrupted left arch. Surgical correction was performed at the age of 3 months. The patient was also noted to have had an ipsilateral large infantile haemangioma. These findings raised the suspicion of the diagnosis of PHACE syndrome. PHACE syndrome is an acronym for Posterior fossa abnormalities, Haemangioma, Arterial anomalies, Cardiac anomalies and Eye anomalies. Future research is needed to elucidate the underlying pathophysiology in PHACE syndrome.

## 1 Patient presentation and past medical history

**Fig. 1:**
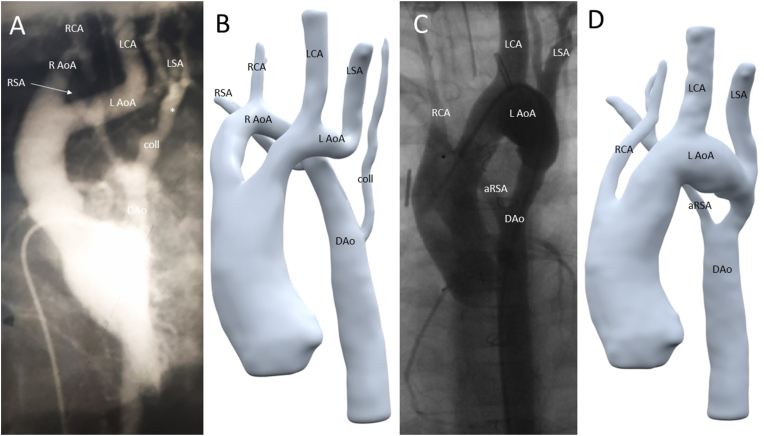
Figure 1: A, B: Angiography (A) and 3D model (B) of preoperative anatomy: right sided aortic arch (R AoA) with right carotid artery (RCA) and right subclavian artery (RSA) connecting towards the descending aorta (DAo). Left sided aortic arch (L AoA) with left carotid artery (LCA) and left subclavian artery (LSA) and interruption thereafter. In addition a collateral vessel (coll) possibly from the vertebrobasilair system to the descending aorta is observed. The interruption of the left-sided arch is indicated by an asterisk (*). C/D: Angiography (C) and 3D model (D) of postoperative anatomy: single remaining left sided aortic arch (L AoA) with right carotid artery (RCA), left carotid artery (LCA), left subclavian artery (LSA) and anomalous right subclavian artery (aRSA).

An 18-year old female visited the outpatient clinic for adults with congenital heart disease. She was born with a double aortic arch, with an interrupted left-sided arch distal from the left subclavian artery and a persisting right-sided arch with a separate origin of the right carotid and subclavian arteries (Fig. 1A and B, online animation https://skfb.ly/oCC9M). In addition there was a large collateral vessel, possibly from the vertebrobasilar system to the descending aorta [1,2]. At the age of three months, she underwent surgical repair anastomosing the left-sided aortic arch to the descending aorta with flap-plasty technique using the collateral vessel and an additional pericardial patch angioplasty. The right-sided aortic arch (specifically between the right carotid artery and right subclavian artery) was described in the surgical charts as “obliterating“ and was transsected to prevent the development of a vascular ring. This left the right-sided subclavian artery in arteria lusoria configuration, deriving from the descending aorta distal to area of aortaplasty (Fig. 1C and D online animation https://skfb.ly/oCC9J).

Five years postoperatively, transthoracic echocardiography suggested a peak gradient of 30 mmHg at the level of the anastomosis. Diagnostic catheterization revealed a hemodynamically insignificant rest-stenosis with a gradient of 10 mmHg.

## 2 History and physical examination

The patient reported no symptoms besides atypical chest pain. Her blood pressure was non-invasively measured as 145/75 mmHg on the left arm. The blood pressure was unmeasurable on the right arm. She had remarkably strong pulsations of the left carotid and the left femoral arteries, while pulsations on the right side were diminished on palpation. She had no dysmorphic features and there was no apparent left-right asymmetry in the development of the extremities.

## 3 Differential diagnostic considerations

The unmeasurable blood pressure on the right arm could have been attributed to a stenosis of the segment between the left subclavian artery and the aberrant right subclavian artery after aortaplasty. However, the differences in pulsations in the left and right femoral arteries could not be explained by a stenosis. She therefore underwent further investigation focusing on detection of a right-sided vascular problem.

## 4 Investigations

**Fig. 2:**
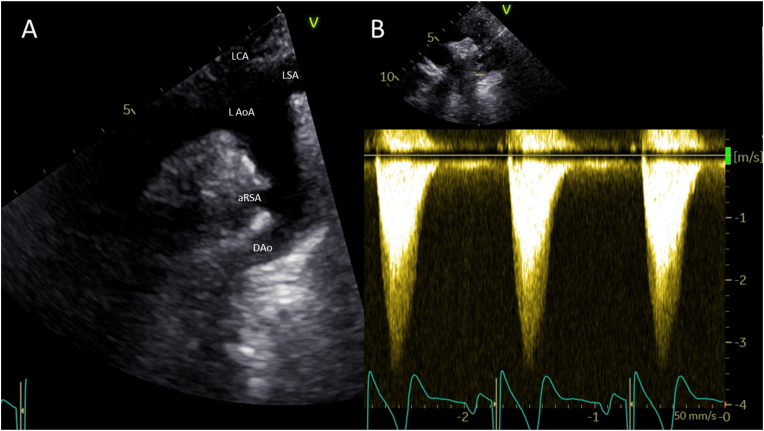
Figure 2: Transthoracic echocardiography of the aortic arch. A: 2 dimensional image of the arch (aRSA: anomalous right subclavian artery, DAo: descending aorta, L AoA: left-sided aortic arch, LCA: left carotid artery, LSA: left subclavian artery). B: Continuous Wave Doppler signal in descending aorta with peak forward flow rate of 3.5 m/s (gradient 49 mmHg) without diastolic run-off.

**Fig. 3:**
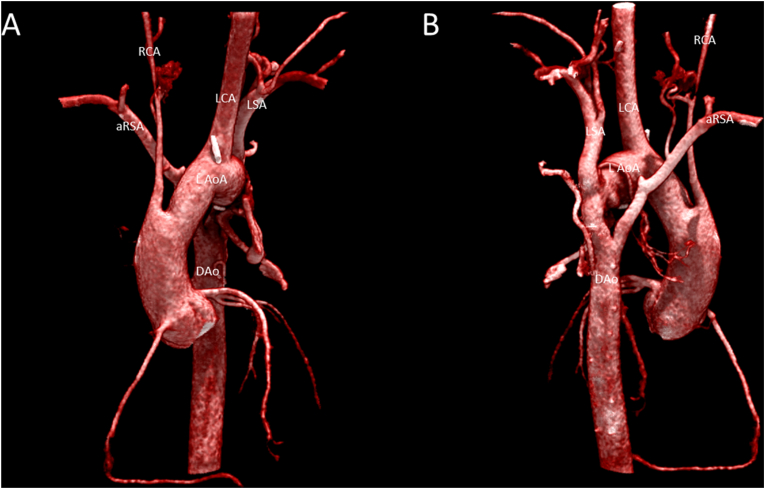
Figure 3: Anterior (A) and posterior (B) view of a cinematic 3D rendering of Computed Tomography Angiography illustrating the aortic arch with hypoplastic right carotid artery and anomalous right subclavian artery. (aRSA: anomalous right subclavian artery, DAo: descending aorta, L AoA: left-sided aortic arch, LCA: left carotid artery, LSA: left subclavian artery, RCA: right carotid artery).

**Fig. 4:**
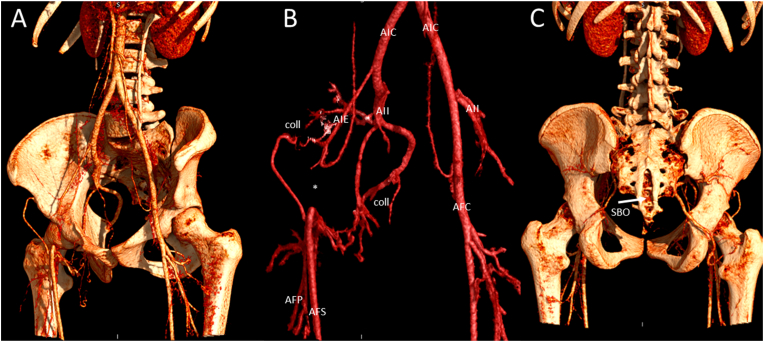
Figure 4: Left anterior oblique view (A and B) and posterior view (C) of a cinematic 3D rendering of Computed Tomography Angiography illustrating a hypoplastic right external iliac artery (AIE) and interrupted right common femoral artery (*) with extensive arterial collaterals (coll) and spina bifida occulta (SBO).

A 24-h ambulatory blood pressure registration on the left arm ruled out hypertension with a mean blood pressure of 126/77 mmHg. Transthoracic echocardiography showed good biventricular function and flow acceleration of up to 3.5 m/s in the descending aorta without diastolic run-off on continuous wave Doppler at the suprasternal notch view (Fig. 2). Computed Tomography Angiography (CTA) revealed a stenotic segment of the proximal descending aorta with a minimum diameter of 10 mm (Fig. 3), corresponding to the site of aortaplasty. Additional Magnetic Resonance Imaging (MRI) with flow analysis revealed a maximum gradient of 27 mmHg over the stenotic segment. The anomalous subclavian artery arose from the descending aorta just distally from the level of stenosis, at a 130⁰ angle relative to the direction of flow. Initial antegrade flow in the anomalous subclavian artery was reversed in late-systole, resulting in a net forward flow of 2 ml/beat, explaining the unmeasurable blood pressure on the right arm. Furthermore, a marked hypoplasia of the right carotid, vertebral, iliac and femoral arteries (the latter with collateral circulation) was noted (Fig. 3, Fig. 4). In addition, a saccular aneurysm of the cavernous segment of the left internal carotid artery and multiple right sided cranial hypoplastic arteries with a well-developed circle of Willis were detected, as well as a persistent stapedial artery on the right side (not shown).

## 5 Management

**Fig. 5:**
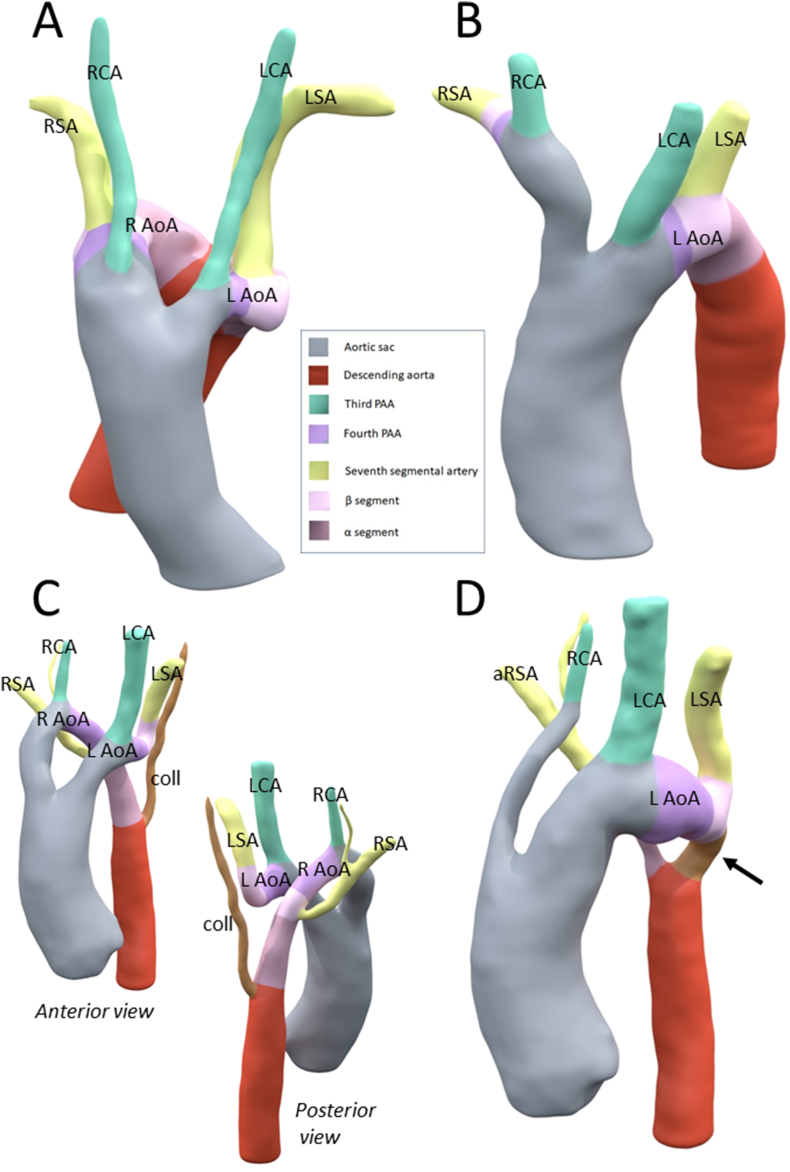
Figure 5: Embryology of the aorta and tributaries. Embryological segments from which the different aortic components have originated, are color-coded according to the panel in the middle. A: 3D reconstruction showing an example of a double aortic arch (left-sided aortic arch (L AoA) and persisting right-sided aortic arch (R AoA), with left- and right-sided carotid artery (LCA and RCA) and left- and right-sided subclavian artery (LSA and RSA). B: Normal left aortic arch anatomy. In this case, a normal regression of the right-sided aortic arch has occurred, with persistence of a left-sided aortic arch (L AoA) with brachiocephalic trunk (nourishing the right subclavian artery (RSA) and right carotid artery (RCA),and a separate origin of the left carotid artery (LCA) and left subclavian artery (LSA). C,D: 3D reconstructions of the pre- and postoperative anatomy of the presented case, on which the embryological segments from which the different segments of the aorta have originated are superimposed by colors. C: preoperative anatomy (from anterior and posterior): double aortic arch with left-sided aortic arch (L AoA) with left carotid artery (LCA), left subclavian artery (LSA) and aortic interruption at the level of the alpha segment (beyond the LSA). Right-sided aortic arch (R AoA) with right carotid artery (RCA) and right subclavian artery (RSA). Collateral vessel (coll) possibly from the vertebrobasilar system to the descending aorta. D: postoperative anatomy: the left-sided arch interruption is restored by using the collateral vessel and a pericardial patch augmentation plasty (arrow). The rightsided aortic arch has been transsected between the right carotid artery (RCA) and the right subclavian artery (RSA) resulting in an anomalous course of the RSA (aRSA). Color coding derived from Ref. [3].

A CTA-based three-dimensional print of the aorta was constructed to facilitate a better understanding of the anatomy and embryology (Fig. 5, online animations https://skfb.ly/oyYxo and https://skfb.ly/oyYxp). The patient was discussed in a multidisciplinary team consisting of adult congenital cardiologists, radiologists, paediatric cardiologists and congenital cardiothoracic surgeons. The gradient in the descending aorta was considered stable over the years and in the absence of hypertension did not warrant intervention. The neurosurgical team advised follow-up imaging for the 3 mm saccular aneurysm in the distal left internal carotid artery. In conclusion, a conservative wait-and-see approach was pursued.

## 6 Embryological considerations

The double arch anatomy with left sided aortic interruption observed in the patient indicates abnormal remodelling of the embryonic aorta arch system. During normal embryology, initially the vascular system has a symmetrical configuration and a double aortic arch system is present until the fifth week of gestation, after which a complex process of remodelling will cause the right-sided arch to gradually disappear. Research in animal models has contributed to our understanding of aortic remodelling during development [3]. In Fig. 5A and online animation https://skfb.ly/oyYxo, an example of a double arch anatomy as related to the embryological segments is shown. Fig. 5B and online animation https://skfb.ly/oyYxp indicate the embryological origin of the arch segments and aortic tributaries in case of a normal left-sided aortic arch (colour coding derived from Ref. [3]). Briefly, the primitive aorta initially consists of 2 ventral aortae that are proximally continuous with the aortic sac (indicated in grey), and of 2 dorsal aortae that are distally continuous with the descending aorta (indicated in red) [3,4]. Between the ventral and dorsal aorta, five paired pharyngeal arch arteries (PAA) develop, of which only the third, fourth and sixth will contribute to the cardiovascular system. The third PAAs (indicated in green) will contribute to the common and internal carotid artery on both sides. The left fourth PAA will form part of the left-sided aortic arch (specifically, the so called B-segment in between the left carotid and the left subclavian artery). On the right side the fourth PAA will initially form part of the right aortic arch and later on (after remodelling by which the right arch disappears) it will form the proximal part of the right subclavian artery (the 4th PAAs are indicated in purple in Fig. 5). The sixth PAA on the left side forms the ductus arteriosus and on the right side it may contribute to the right pulmonary artery. Both subclavian arteries develop from the seventh segmental artery and the alpha segment of the right dorsal aorta normally regresses [3,5] In our case (Fig. 5C, online animation https://skfb.ly/oyYxu), the embryologic double arch anatomy persisted, although a regression of the left sided 4th PAA seems to have occurred after birth, causing the right-sided aortic arch to obliterate between the right carotid and right subclavian artery (the so called B-segment). In addition, there was an abnormal regression of the left sided alpha segment, causing to the patients’ left sided interruption of the aorta. Fig. 5D and online animation https://skfb.ly/oyYxw illustrate the situation after surgery.

## 7 Diagnostic considerations at follow-up and genetic counselling

During follow-up, it was revealed that the patient had initially presented at the age of 3 months with a large right sided facial haemangioma. At that age, in order to perform an MRI under sedation to exclude an associated Dandy Walker malformation, the patient had been referred to the anesthesiologist who auscultated a precordial murmur. Subsequently, the aortic arch abnormality was diagnosed. Cerebral MRI was unremarkable at that time and the haemangioma regressed spontaneously during follow-up.

**Fig. 6:**
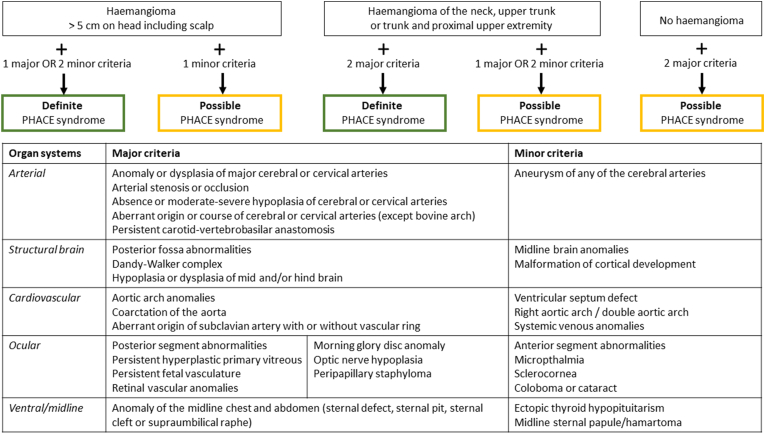
Figure 6: Diagnostic criteria for PHACE syndrome. Adopted from Garzon et al. [6].

The combination of the haemangioma, arterial hypoplasia and aortic arch abnormality may be a part of the PHACE syndrome. PHACE syndrome is an acronym for Posterior fossa abnormalities, Haemangioma, Arterial anomalies, Cardiac anomalies and Eye anomalies [6]. PHACE syndrome is a rare disorder, first described in 1996 [7,8]. The diagnostic criteria have been updated in 2016 (Fig. 6) [6]. The diagnosis can be made definitely in patients with a facial haemangioma of >5 cm in diameter and meeting one major criterium or two minor criteria. The associated arterial malformations in PHACE syndrome are usually on the ipsilateral side of the haemangioma [8]. The observed cardiovascular malformations are most often aortic arch anomalies with a coarctation, double or interrupted aortic arch, as was also observed in our patient [9]. The arch abnormalities in PHACE syndrome are distinct in location and character (multiple long segments of narrowing) compared to a classical focal coarctation at the aortic isthmus. Notable is the absence of associated left heart valve abnormalities (such as bicuspid aortic valve) in contrast to a classical coarctation. In the current era, the aortic arch and the posterior fossa anomalies are typically identified during routine fetal ultrasound examination.

The pathophysiology of PHACE syndrome is largely unknown. Striking is the female preponderance with reported male-to-female ratios of 1:4.6–9^7,9^. Several genetic studies have been performed to identify the cause of PHACE syndrome, including X-inactivation studies, copy number variation, and somatic mutation analysis. So far, the exact genetic cause remains unknown. In addition, there has been no recurrence described in families [10]. Typically, one or two structural anomalies are present in addition to the haemangioma; whereas the full phenotype is rarely present. Female development in utero generally lags behind male development, which may result in insufficient blood supply and oxygenation of the developing brain. Disturbances in neural crest cell migration might play a role in the pathogenesis of PHACE syndrome [10], although further research is necessary to elucidate the exact aetiology.

The present case had a large right-sided facial haemangioma and met two major criteria (i.e. arterial and cardiovascular anomalies, Fig. 6). The patient was referred to the clinical geneticist and the diagnosis PHACE syndrome was confirmed. The arterial anomalies consisted of multiple hypoplastic cerebral arteries on the right side and a persisting right stapedial artery (remnant of the 2nd pharyngeal arch artery). The cardiovascular anomaly consisted of a double aortic arch of which the left-sided arch was interrupted. Coincidentally, during follow up a medialisation of the vocal cords was observed, due to reduced motility of the right vocal cord, with intact vocal ability. This suggests either malfunction or maldevelopment of the right laryngeal recurrent nerve. This is considered to be related to previous surgery, although a developmental origin cannot be excluded [11]. In addition to the above-mentioned anomalies, she had a hypoplastic right external iliac artery and occlusion of the right common femoral artery, which has not previously been described as part of the PHACE syndrome. Unilateral and bilateral iliofemoral artery hypoplasia is rarely described and is often an incidental finding [12]. Lastly, a spina bifida occulta was found coincidentally (Fig. 4C). This has also not previously been described in PHACE syndrome, however, midline defects such as a sternal cleft have previously been associated with PHACE syndrome [7,8]. From an embryological point of view, midline defects as well as cardiac and outflow tract/arterial anomalies are considered to be related to neural tube and/or crest defects [13].

## 8 Follow-up

Current guidelines recommend long-term annual follow-up after surgery for coarctation. Imaging of the aorta to document post-repair anatomy and potential complications (re-coarctation or aneurysm formation) should be performed every 3–5 years, preferably with MRI. In case of arterial hypertension and a peak-to-peak gradient of >20 mmHg, reintervention is indicated [14].

## 9 Conclusion

The presented case describes a patient with a double aortic arch with interruption of the left-sided aortic arch and remarkable hypoplasia of the right-sided arterial system. It was especially noteworthy that this patient had no dysmorphic features and that there was no left-right asymmetry in the development of the extremities. During further follow up, the combination of large infantile haemangioma, hypoplastic right-sided cerebral arteries, interrupted left-sided aortic arch and persistent right-sided aortic arch, was recognised to fulfil the criteria of the PHACE syndrome. Considering the new diagnostic criteria for PHACE syndrome, more patients might meet the criteria in contrast to what has previously been reported. Future research is needed to elucidate the underlying pathophysiology in PHACE syndrome and to define whether long-term follow-up should differ from the follow-up of classical coarctation.

## Learning objectives

- Infantile haemangioma can be associated with arterial and cardiac anomalies and be seen as part of the PHACE syndrome.
- Three dimensional reconstruction of CTA imaging can be helpful in understanding complex aortic arch anatomy.

## Declaration of competing interest

The authors declare that they have no known competing financial interests or personal relationships that could have appeared to influence the work reported in this paper.

The patient gave her consent regarding this publication.
